# Effect of Adherence to Physical Exercise on Cardiometabolic Profile in Postmenopausal Women

**DOI:** 10.3390/ijerph18020656

**Published:** 2021-01-14

**Authors:** Valentina Bucciarelli, Francesco Bianco, Francesco Mucedola, Andrea Di Blasio, Pascal Izzicupo, Desiree Tuosto, Barbara Ghinassi, Ines Bucci, Giorgio Napolitano, Angela Di Baldassarre, Sabina Gallina

**Affiliations:** 1 Department of Neurosciences, Imaging and Clinical Sciences, “G. d’Annunzio” of Chieti-Pescara, Via dei Vestini, 66100 Chieti, Italy; dr.francescobianco@gmail.com (F.B.); fra.mucedola@gmail.com (F.M.); desiree.tuosto@hotmail.it (D.T.); sgallina@unich.it (S.G.); 2 Department of Medicine and Aging Sciences, University “G. d’Annunzio” of Chieti-Pescara, Via dei Vestini, 66100 Chieti, Italy; andiblasio@gmail.com (A.D.B.); izzicupo@unich.it (P.I.); b.ghinassi@unich.it (B.G.); ines.bucci@unich.it (I.B.); gnapol@unich.it (G.N.); a.dibaldassarre@unich.it (A.D.B.)

**Keywords:** adherence to physical exercise, gender medicine, menopause, cardiovascular risk, sedentary time

## Abstract

*Background*: Menopause is associated with negative cardiovascular adaptations related to estrogen depletion, which could be counteracted by physical exercise (PhE). However, the impact of total adherence-rate (TA) to PhE and sedentary time (SedT) on cardiometabolic profile in this population has not been elucidated. *Methods:* For 13-weeks, 43 women (57.1 ± 4.7 years) participated in a 4-days-a-week moderate-intensity walking training. They underwent laboratory, anthropometric and echocardiographic assessment, before and after training (T0–T1). Spontaneous physical activity (PhA) was assessed with a portable multisensory device. The sample was divided according to TA to PhE program: <70% (*n* = 17) and ≥70% (*n* = 26). *Results:* TA ≥ 70% group experienced a significant T1 improvement of relative wall thickness (RWT), diastolic function, VO2max, cortisol, cortisol/dehydroandrostenedione-sulphate ratio and serum glucose. After adjusting for SedT and 10-min bouts of spontaneous moderate-to-vigorous PhA, TA ≥ 70% showed the most significant absolute change of RWT and diastolic function, body mass index, weight and cortisol. TA ≥ 70% was major predictor of RWT and cortisol improvement. *Conclusions:* In a group of untrained, postmenopausal women, a high TA to a 13-weeks aerobic PhE program confers a better improvement in cardiometabolic profile, regardless of SedT and PhA levels.

## 1. Introduction

Cardiovascular disease (CVD) represents the leading cause of morbidity and mortality in women [1]. Substantial gender differences in the development of CVD exist, mainly related to the protective anti-inflammatory and anti-apoptotic activity of estrogens [2].

Besides traditional cardiovascular (CV) risk factors, there is an emerging category of risk factors specific for the female gender, including autoimmune disease, breast cancer treatment, cardiometabolic gestational disorders and menopause [3].

The decline in ovarian hormone concentrations during the menopausal transition and beyond seems to be involved in the development of CVD in women, as estrogens play a relevant role in endothelial function, vascular tone and cardiac function, as well as in lipid profile and inflammatory status [4].

Postmenopausal women exhibit an exponential increase in the incidence of ischemic heart disease, mostly related to microvascular dysfunction and abnormal coronary reactivity, and heart failure with preserved ejection fraction, as compared with men of the same age [5,6]. In this regard, strong evidence exists about the impact of estrogen depletion both on early diastolic relaxation and late diastolic compliance, leading to left ventricular diastolic dysfunction, fibrosis and concentric left ventricular hypertrophy, namely CV remodeling associated with menopause transition [7,8,9].

On the other hand, exercise training has been described as a powerful tool to counteract structural and functional cardiac changes in CVD, by contributing to the phenotypical changes of pathological into physiological cardiac hypertrophy. Moreover, it reduces the body mass index and improves insulin sensitivity as well as glucose uptake and lipid profile [10]. Although these effects have been largely confirmed in postmenopausal women, there is still poor worldwide adherence to World Health Organization Recommendation about physical activity (PhA), especially in female population [11,12].

Although physical activity (PhA) plays a paramount role in reducing CVD-related incidents, recent data from clinical trials suggest that activity alone is not enough to reduce the risk of CVD in older adults. Both physical inactivity (PhI), defined as insufficient PhA level to meet present PhA recommendations for age, and sedentary behavior (SedB), defined as any waking behavior characterized by an energy expenditure ≤1.5 metabolic equivalents (METs), while in a sitting, reclining or lying posture, have been recently identified as having negative impacts on health status in older adults [13,14]. PhI is considered a major risk factor for global mortality and CVD, being greatly associated with severe menopausal symptoms and obesity in postmenopausal women [15]. Similarly, SedB has been associated with metabolic disorders, CVD, cancer, mortality, and psychological distress. Therefore, reducing sedentary activity provides an alternative strategy to reduce the risk of CVD and CVD-related mortality among older adult [16]. Ekelund et al. recently showed that all intensities of PhA, including light intensity, are associated with a substantially reduced risk of death in a dose-response manner, with a statistically significant higher risk of death was observed for sedentary times of 9.5 or more hours daily [17]. Moreover, even moderate intensity PhA does not eliminate the increased risk associated with SedB [18].

On the other hand, adherence represents another crucial key point potentially affecting health goals related both to PhA and physical exercise (PhE), the latter defined as a subcategory of PhA that is planned, structured, repetitive, especially in older adult population [19]. Exercise adherence is generally defined as successful if participants complete a prescribed exercise routine for at least two-thirds of the time [20].

Among clinical trials assessing the role of exercise adherence on CV outcomes, efficacy trials report a high adherence-rates (TA) to PhE (75–85%), but the impact of lower TA on cardiometabolic status has not been established yet, especially in postmenopausal women, which could most benefit from PhE.

Given these premises, we hypothesized that the adherence-rate to PhE may be able to influence and modulate the beneficial effects associated to PhE itself. Thus, our aim was to assess the impact of high and low adherence-rates to PhE on metabolic status and CV remodeling in a population of untrained, postmenopausal women, without history of any CV or any other systemic chronic disease, utilizing transthoracic echocardiography, laboratory and anthropometric parameters. Moreover, we evaluated the potential interactions between TA, sedentary time (SedT) and cardiometabolic profile.

## 2. Materials and Methods

### 2.1. Study Protocol and Population

This is an observational, cross-sectional study in which we screened a cohort of untrained, postmenopausal women who voluntarily joined our program from a public advertisement placed in the outpatients’ clinic of different general practitioners from the area of Pescara (Italy). One hundred and thirty-four women (mean age 57.1 ± 4.7 years) adhered to our screening. Participants’ medical history was assessed during a telephone interview; in particular, we asked for any history of cardiovascular, pulmonary, endocrine, orthopedic or systemic disease, chronic medical therapy, and previous hospitalizations for any cause within 5 years from the interview. The postmenopausal state was defined as a period of at least 12 months of amenorrhea, with plasma estradiol levels < 20 pg/mL. The untrained state was defined as no attendance to a PhE program in the previous two years. After the evaluation of medical history, participants underwent a preliminary CV assessment, including both transthoracic echocardiogram and exercise stress test. Then, applying our inclusion/exclusion criteria, we enrolled 43 postmenopausal women.

The inclusion criteria were: age < 65 years, body mass index (BMI) between 18.5 kg/m^2^ and 40 kg/m^2^; no attendance at PhE or adherence to controlled-dietary-plans in the previous two years; no history of endocrine, pulmonary or cardiovascular disease; no history of diabetes mellitus, smoking habit, systemic arterial hypertension nor dyslipidemia; no use of any medications or dietary supplements; no history of orthopedic or systemic diseases potentially limiting the walking training; unremarkable medical evaluation and preliminary CV assessment. The examinees, which did not meet the inclusion criteria, and presented a pathological medical evaluation or atypical exercise stress test results, were excluded from the study.

**Figure 1:**
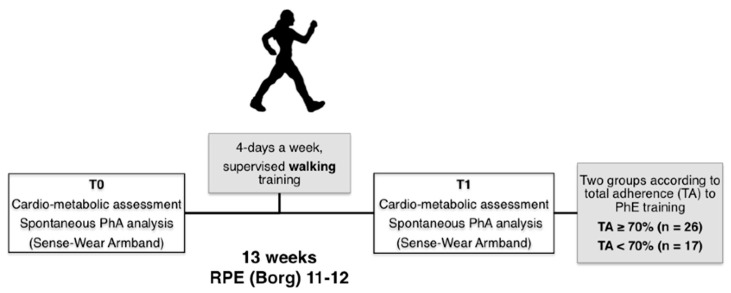
Flow-chart of study protocol. Abbreviations: PhA, physical activity; RPE, rated perceived exertion; TA, total adherence; T0, pre-training; T1, post-training.

Then, the participants underwent laboratory, anthropometric and CV assessment, including both transthoracic echocardiogram and exercise stress test, before (T0) and after training (T1). Moreover, the analysis of their daily physical activity was made with a portable motion sensor (Sense-Wear SenseWear Pro2 Armband, BodyMedia, Pittsburgh, PA, USA) (Figure 1).

The Ethical Committee of our institution approved the study protocol (deliberative act n.1070, 24 October 2013). The participants gave written informed consent before the analysis.

### 2.2. Variable Definitions

For our purposes, we defined our variables, as shown below:

- Total adherence (TA): percentage of total ExV performed on planned total ExV;
- Cardiovascular profile: blood pressure (BP; systolic blood pressure, SBP; diastolic blood pressure, DBP; mean blood pressure, MBP) and heart rate (HR); left ventricular mass index (LVMi); left ventricular (LV) systolic and diastolic function; left atrium diameter (LAD); aortic distensibility (AD); maximal oxygen uptake (VO2max);
- Metabolic profile: total cholesterol level (TC), high-density lipoprotein cholesterol (HDL-C), low-density lipoprotein cholesterol (LDL-C), triglycerides (Tg); fasting glucose levels; leptin; cortisol; dehydroepiandrosterone sulfate (DHEAS); cortisol-to-DHEAS ratio (cortisol/DHEAS); fat mass (FM); leptin-to-fat mass ratio (leptin/FM); waist circumference (WC) and hip circumference (HC).

### 2.3. Anthropometric Assessment, Blood Sampling and Cardiovascular Eligibility

Bodyweight, stretched height, waist circumference (WC) and hip circumference (HC) were assessed according to the International Society for the Advancement of Kinanthropometry’s guidelines. Body weight and stretched stature were measured to the nearest 0.1 kg and 0.1 cm, respectively, with the participants dressed in light clothing and without shoes, using a stadiometer with a balance-beam scale (Seca 220; Seca, Hamburg, Germany). Body mass index (BMI) was calculated according to the formula of body weight/stature^2^ (in kilograms per meter squared), whereas body surface area (BSA) was assessed according to the formula by Du Bois et al. [21]. An anthropometric tape (Seca 200) was used to measure WC and HC. WC was measured as the smallest circumference between the rib cage and the iliac crest, at the end of normal expiration, whereas HC was measured at the level of the widest circumference between the waist and the thighs [22]. The body composition, in particular the fat mass variations before and after training, was analyzed using electrical bioimpedance (BIA; Tanita BC—420MA Tanita, Tokyo, Japan). After 12 h overnight fasting, venous blood samples were collected to assess serum lipid profile (total cholesterol, TC; low-density lipoprotein cholesterol, LDL-C; high-density lipoprotein cholesterol, HDL-C; triglycerides, Tg), leptin, fasting glucose levels, leptin, cortisol and dehydroepiandrosterone sulphate (DHEA-S).

Fasting glucose levels, HDL-C, TC, and Tg were assessed through enzymatic methods, with LDL-C calculated according to the Friedewald equation. Enzyme-linked immunosorbent assay was used to measure leptin (DBC Inc., London, ON, Canada), cortisol and DHEA-S (DRG Instruments GmbH, Marburg, Germany).

Systemic blood pressure (BP) comprehensive of systolic blood pressure (SBP), diastolic blood pressure (DBP) and mean blood pressure (MBP) was measured twice, using a mercury sphygmomanometer (Erkameter 3000, Erka, Bad Tolz, Germany) after 5 min of seated rest, with 1–2 min pause between each measurement. The average of the measurements was used as the baseline BP. A 12-lead electrocardiogram (ECG) (P8000 Esaote, Italy) was assessed before the aerobic fitness test to register basal heart rate (HR), with subjects standing supine for 10 min. Both fitness level and eligibility for aerobic training were assessed through a graded maximal exercise test on a cycle ergometer (SANA BIKE 150 F, Ergosana GmbH, Bitz, Germany). Participants were tested under continuous ECG monitoring (AT-10 plus, SCHILLER, Baar, Switzerland) and step-by-step blood pressure measurement. Maximal oxygen uptake (VO2 max) was estimated by multiplying the maximal MET by 3.5.

All the patients underwent a complete transthoracic echocardiographic study (M-mode, 2-dimensional and Doppler measurements). The exams were performed using a 3.7 MHz electronic probe (GE Vivid E, GE Medical Systems, Milwaukee, WI, USA). Images were stored digitally and analyzed offline, according to the International Guidelines from the American Society of Echocardiography/European Society of Echocardiography [23]. In particular, we focused on the following parameters:

- M-mode parameters: interventricular septum thickness at end-diastole (IVSd, mm), interventricular septum thickness at end-systole (IVSs, mm), left ventricular internal dimension at end-diastole (LVIDd, mm), left ventricular internal dimension at end-systole (LVIDs, mm), posterior wall at end-diastole (PWd, mm), posterior wall at end-systole (PWs, mm), left ventricular mass (LVM, g), left ventricular mass indexed to allometric height in meters raised to the power of 2.7 (LVMi, g/m^2.7^), left ventricular mass indexed to body surface area (LVMi/BSA), relative wall thickness (RWT), left atrial diameter (LAD, mm), tricuspid annular plane systolic excursion (TAPSE, mm);
- 2-dimensional parameters: left ventricular end-diastolic volume (LVEDV, mL) and LVEDV indexed for BSA (LVEDV/BSA), left ventricular end-systolic volume (LVESV, mL) and LVESV indexed for BSA (LVESV/BSA), left ventricular ejection fraction (LVEF, %);
- Doppler measurements: left ventricular diastolic function assessment (E/A ratio, E/e’).

We assessed aortic distensibility (AD) as previously described [24].

### 2.4. Daily Physical Activity Assessment

Daily PhA was measured under free-living conditions over five consecutive days, including four weekdays and one weekend day, employing SenseWear Proarmbands (BodyMedia, Pittsburgh, PA, USA), before (T0) and after (T1) exercise training. Among the whole recorded data, we focused our attention on the time spent in PhA with an intensity of ≤1.5 METs, excluding nocturnal sleeping (sedentary time, SedT) and the time spent in physical activities >1.5 METs and <3 METs (low-intensity physical activity, LIPAT), as well as the time in physical activities >3 METs and <6 METs (moderate-intensity physical activity, MIPAT). Moreover, the average of daily energy expenditure (DEE), MET and steps, as well as 10-min bouts of spontaneous moderate-to-vigorous physical activity (Bout10) were considered. Participants carried their wearable monitors all days, except while bathing. The wear time criteria, to consider valid registrations, were at least 540 min/day on weekdays and 480 min/day on weekend days.

### 2.5. Exercise Training Program

The participants worked out at moderate intensity for 13 weeks, 4-days a week. Exercise intensity was dispensed and monitored, as stated in the ratings of the perceived exertion method [25]. The study was conducted in summer, to eliminate the effect of the seasonal changes on PhA [26].

During the first month, each training session lasted 40 min, with a walking velocity eliciting an effort equal to 11 according to the 15-category rating of perceived exertion scale (RPE) [27]. During the second month, each training session lasted 50 min, with the same walking velocity. During the third month, participants increased only the training intensity from 11 to 12 RPE. Compliance with the training sessions was checked through both women’s and exercise trainers’ diaries. Walking training was the only study intervention. We calculated the volume of the completed exercise session, multiplying the time of the session for the RPE point. The amount of all exercise sessions gave the size of the exercise program of each participant.

For each work out session, exercise volume (ExV) was estimated as (exercise minutes * RPE). Total ExV was calculated as the sum of each work out ExV. Total adherence (TA) was determined as the percentage of total ExV performed on planned total ExV. We retrospectively divided the participants in two groups according to TA (group 1 [*n* = 26]: TA ≥ 70%; group 2 [*n* = 17]: TA < 70%). The mean TA (±SD) in group 1 and 2 were, respectively, 89.37 ± 9.9% and 48.4 ± 16.28%.

### 2.6. Statistical Analysis

At the best of our knowledge, the two largest reports that evaluated the effects of physical exercise programs on cardiometabolic profile in postmenopausal women enrolled respectively 31 and 41 women [28,29]. Therefore, we assessed adequate a population size of 30 patients (confidence level = 95%, margin of error = 5%) to reach a sufficient statistical power [30]. Continuous variables are presented as mean and standard deviation (SD) or median and lower/upper limit (Q1, Q3); while, categorical data as absolute numbers and percentages, as appropriate. Adjusted means and standard errors (SE) derived from linear/logistic regression models. All the variables were tested for normality using the Shapiro–Wilk test.

For our purposes, we divided the participants into two groups according to TA of PhE: <70% and ≥70%; then, we estimated the absolute change for each variable, using the following formula: post-exercise program value minus the pre-exercise program value divided by pre-exercise program, in order to normalize for T0 values all the variables. Differences between groups and absolute changes were assessed utilizing the Student’s t-test or a non-parametric t-test, according to their distribution.

Correlations between the TA of PhE and the absolute changes were calculated through a multivariable regression model, adjusted for age, SedT and Bout10.

A two-tailed *p*-value of 0.05 was considered statistically significant. Statistical analysis was performed using the SPSS software package (SPSS 22.0, Chicago, IL, USA) and Prism 6.0 (GraphPad Software, La Jolla, CA, USA).

## 3. Results

### 3.1. Baseline Characteristics

The baseline characteristics of our population are shown in Table 1.

### 3.2. Cardiovascular Profile

In the whole population, we observed a significant reduction in HR (*p* < 0.001), SBP (*p* = 0.02), DBP (*p* = 0.01) and MBP (*p* = 0.01) at the end of the training program (T1). Echocardiographic results showed significant changes in interventricular septum thickness at end-systole (IVSs, *p* = 0.01), left ventricular internal dimension at end-diastole (LVIDd, *p* = 0.003), left ventricular mass (LVM, *p* = 0.03), left ventricular mass indexed (LVMi, g/BSA, *p* = 0.04), relative wall thickness (RWT, *p* = 0.04), E/A ratio (*p* = 0.01), E/e’ (*p* = 0.04), aortic distensibility (AD, *p* = 0.005) (Table 1).

After dividing the population as for TA, we found in both groups a significant reduction in heart rate (TA ≥ 70%, *p* = 0.003; TA < 70%, *p* = 0.002). On the other hand, only TA ≥ 70% group showed an improvement into RWT (*p* = 0.01), E/A ratio (*p* = 0.01) and E/e’ (*p* = 0.01) (Table 2). At the analysis of delta absolute change, adjusted for age, SedT and Bout10, the TA ≥ 70% group showed the most significant absolute change of RWT and E/e’ (*p* = 0.038 and *p* = 0.039, respectively) (Table 3). At the multivariable analysis, TA ≥ 70% was the only predictor of improvement in RWT (Table 4).

### 3.3. Metabolic and Anthropometric Profile

In the whole study population, we found a significant reduction in cortisol (*p* < 0.001), cortisol-to-DHEAS ratio (*p* = 0.01), leptin (*p* < 0.001), the leptin-to-fat mass ratio (*p* < 0.001) and fasting glucose levels (*p* = 0.002). We also observed a significant reduction both in HC (*p* < 0.001). VO2max significantly increased after physical training (*p* = 0.007) (Table 1).

After dividing the population as for TA, we found in both groups a significant reduction in hip circumference (HC, TA ≥ 70%, *p* = 0.003; TA < 70%, *p* = 0.005) and leptin (TA ≥ 70%, *p* < 0.0001; TA < 70%, *p* < 0.001). On the other hand, only TA ≥ 70% group showed an improvement into VO_2_max (*p* = 0.007), cortisol (*p* = 0.016) and serum glucose (*p* = 0.02) (Table 2). At the analysis of delta absolute change, adjusted for age, SedT and Bout10, the TA ≥ 70% group showed the most significant absolute change of BMI (*p* = 0.046), weight (*p* = 0.046) and cortisol (*p* = 0.041) (Table 3). At the multivariable analysis, TA ≥ 70% was the only predictor of improvement in cortisol (Table 4).

### 3.4. Spontaneous Physical Activity Analysis

At T1, we observed a significant increase in daily energy expenditure (DEE avg, *p* < 0.001). We found no differences in SedT, as well in LIPAT and MIPAT, at T1 as compared to T0 (Table 5).

At the multivariable analysis, TA ≥ 70% was the only predictor of improvement both in RWT and cortisol (Table 4).

## 4. Discussion

In our population of postmenopausal, untrained women, we demonstrated a significant cardiometabolic improvement at the end of PhE training (T1), with a reduction of HR and BP, LV remodeling toward eccentric hypertrophy, as well as the increase in both diastolic function, evaluated as the Doppler-derived E/A ratio, E/e’ and AD. Moreover, we found an important decrease in cortisol, cortisol-to-DHEAS ratio, leptin, L/FM and fasting glucose levels, as well as in HC and FM. VO2max significantly improved after physical training. Only the group with TA ≥ 70% showed a considerable delta change for RWT, E/e’, BMI, weight and cortisol, regardless of SedT and Bout10. At the multivariable analysis, TA ≥ 70% was the only predictor of improvement both in RWT and cortisol.

PhE has several positive effects on cardiometabolic profile across the lifespan, and it plays a relevant role in counteracting the unfavorable adaptations related to menopausal transition. Several studies evaluated the impact of different PhE protocols associated with specific dietary intake or vitamin supplements on body composition, lipid and glucidic profile, antioxidant status and prothrombotic burden, and cardiovascular parameters in healthy postmenopausal women [31,32,33,34,35,36,37].

Our results showed that a 13-weeks, aerobic PhE program is able to counteract the effects of estrogen depletion on the CV system, which are considerably associated with the development of arterial stiffness, diastolic dysfunction and heart failure with preserved ejection fraction [38]. Moreover, we observed an improvement in metabolic profile, especially in terms of HC, leptin, leptin/FM and cortisol, the latter being a causal risk factor for CVD, especially in women [39,40].

VO2max significantly increased after PhE program without differences as for TA. Lynch et al. previously showed that postmenopausal women have a lower VO2max than peri-menopausal women of a similar age and adiposity, which may be associated with an increased risk of total and central obesity and CV [41].

Regarding accelerometer parameters, we did not observe any increase in SedT, according to TA.

A negative compensation of non-exercise physical activity in postmenopausal partecipants in exercise training has been described, potentially limiting the beneficial effects of physical exercise program [42]. Finally, at the analysis of delta absolute change adjusted by age, SedT and Bout10, we found the most significant improvement in RWT, E/e’, BMI, weight and cortisol in the group with TA ≥ 70%.

LaMonte et al. analyzed the relationship between LIPAT and CV risk factors, demonstrating that PhA measured by accelerometry, including LIPAT, is associated with lower CV risk factor levels in older women [43].

On the other hand, even high activity levels of PhA do not eliminate the increased risk associated with SedT [18], although the shift from non-prolonged sedentary or LIPAT determines a significant improvement in mean levels of WC and BMI [44].

Our data show that TA to physical exercise plays a relevant role on cardiometabolic remodeling in postmenopausal women. Moreover, in our population this parameter did not negatively affect spontaneous PhA, nor SedT.

The limitations of our study were the small sample size and the limited echocardiographic and vascular measurements taken into account. The absence of a non-exercising group can be considered a study limitation, too. However, we utilized simple and easy-to-obtain parameters to assess, for the first time in literature, the role of adherence to PhE on cardiometabolic remodeling in a population of postmenopausal, untrained women as well as the interaction between TA to PhE, SedT and the amount of cardiometabolic improvement.

## 5. Conclusions

The role of physical exercise on preventing cardiometabolic risk factors has been extensively described; however, little is known about the role of adherence to physical exercise training on cardiometabolic remodeling, especially in postmenopausal women. According to our data, an adherence rate ≥70% to a 13-weeks aerobic supervised physical training is able to improve cardiometabolic parameters, especially in terms of left ventricular remodeling and stress hormone levels, counteracting the negative adaptations associated with menopause and the increase of cardiovascular risk. TA to PhE did not influence compensative lowering of PhA and did not determine an increase in SedT in our population. Our data are in line with the current evidence from cross-sectional and prospective cohort studies. Moreover, we highlighted the need for a call for action to motivate our patients to best complies with PhE programs, achieving the most significant benefit on cardiometabolic risk factors.

## Figures and Tables

**Table 1:** Basal characteristics (pre-training, T0) and post-training (T1) cardiometabolic profile (*n* = 43).

**(a) General and Anthropometric Characteristics**	**Pre-Training T0**	**Post-Training T1**	*p*-Value *
Age (years)	57.1 ± 4.7	-	-
TA, %	72.1 ± 23	-	-
BSA (m^2^)	1.66 ± 0.14	-	-
BMI (Kg/m^2^)	26.1 ± 3.7	26 ± 3.5	0.89
Weight (kg)	65.8 ± 11.7	65.6 ± 11.6	0.93
WC (cm)	82.6 ± 9	82.1 ± 8.6	0.96
HC (cm)	102 ± 8.1	99 ± 7.4	<0.001
FM, kg	23.9 ± 8.6	23.7 ± 8.4	0.91
VO2 max (mL/kg/min)	25.4 ± 5.5	27.6 ± 5.5	0.007
**(b) Laboratory Parameters**	**Pre-Training T0**	**Post-Training T1**	*p*-Value *
DHEA-S (pg/mL)	0.72 [0.54–1.22]	0.86 [0.52–1.47]	0.09
Cortisol (pg/mL)	104.3 [85.7–148.5]	92.5 [66.6–119]	<0.001
Leptin (ng/mL)	45.04 [22.6–70.2]	39.3 [10.1–55]	<0.001
Cortisol/DHEA-S	137.3 [77.2–241.4]	126.6 [48.5–211.8]	0.01
Leptin/FM ratio	2 [1.2–2.9]	1.2 [0.5–2]	<0.001
Serum glucose (mg/dL)	79.5 [73–81]	76.5 [73–81]	0.002
TC (mg/dL)	239 [214–258]	239 [204–261]	0.96
LDL-C (mg/dL)	145.5 [128–168]	144.5 [127–170]	0.74
HDL-C (mg/dL)	62.5 [53–78]	65.5 [53–76]	0.57
Tg (mg/dL)	99.5 [73–137]	92 [62–125]	0.88
**(c) Cardiovascular Parameters**	**Pre-Training T0**	**Post-Training T1**	*p*-Value *
SBP (mmHg)	125 ± 12	121.1 ± 9.1	0.02
DBP (mmHg)	78.2 ± 6.2	75.8 ± 6.5	0.01
MBP (mmHg)	93.8 ± 7.7	90.9 ± 6.8	0.01
HR (bpm)	67.8 ± 7.7	62.7 ± 5.6	<0.001
IVSd (mm)	9.4 ± 1.2	9.5 ± 0.12	0.59
IVSs (mm)	13.5 ± 1.5	14.1 ± 0.15	0.01
LVIDd (mm)	43.3 ± 3.4	45 ± 0.31	0.003
LVIDs (mm)	25.9 ± 4.3	25.5 ± 0.36	0.57
PWd (mm)	8.6 ± 1.1	8.5 ± 0.1	0.55
PWs (mm)	13.5 ± 1.9	14 ± 1.8	0.21
LVM (g)	135.5 ± 35.1	143.6 ± 37.7	0.03
LVMi (g/m ^2.7^)	41.9 ± 12.1	43.8 ± 10.6	0.44
LVMi (g/BSA)	84.8 ± 15.3	89.4 ± 16.7	0.04
RWT	0.4 ± 0.06	0.38 ± 0.04	0.04
E/A ratio	0.92 ± 0.3	1.02 ± 0.25	0.01
E/e’	6.9 ± 1.7	6.1 ± 1.6	0.04
AD (cm^2^dyne^−1^ 10^−6^)	0.22 ± 0.14	0.26 ± 0.14	0.005
TAPSE (mm)	22 ± 2.3	23 ± 3.5	0.12
LAD (mm)	33.4 ± 4.2	34 ± 3.4	0.46
LVEDV (mL)	87.4 ± 9.5	89.5 ± 11.6	0.36
LVEDV/BSA (mL/mq)	52.7 ± 5.4	54 ± 7.4	0.35
LVESV (mL)	28.6 ± 6.6	27.2 ± 7.2	0.35
LVESV/BSA (mL/mq)	17.2 ± 3.6	16.8 ± 4.2	0.63
LVEF (%)	54.8 ± 9.5	57.8 ± 13	0.22

Legend: Data are described as mean ± SD or median (Q1, Q3) for quantitative variables; *: Student’s T-Test or non-parametric *T*-test. Abbreviations: TA, total adherence; BSA, body surface area; BMI, body mass index; kg, kilograms; WC, waist circumference; HC, hip circumference; FM, fat mass; VO2max, maximal oxygen uptake; DHEA-S, dehydroepiandrosterone sulphate; TC, total cholesterol; LDL-C, low-density lipoprotein cholesterol; HDL-C, high-density lipoprotein cholesterol; Tg, triglycerides; SBP, systolic blood pressure; DBP, diastolic blood pressure; MBP, mean blood pressure; HR, heart rate; IVSd, interventricular septum thickness at end-diastole; IVSs, interventricular septum thickness at end-systole; LVIDd, left ventricular internal dimension at end-diastole; LVIDs, left ventricular internal dimension at end-systole; PWd, posterior wall at end-diastole; PWs, posterior wall at end-systole; LVM, left ventricular mass; LVMi (g/m ^2.7^), left ventricular mass indexed to allometric height in meters raised to the power of 2.7; LVMi (g/BSA), left ventricular mass indexed to body surface area; RWT, relative wall thickness; AD, aortic distensibility; TAPSE, tricuspid annular plane systolic excursion; LAD, left atrial diameter; LVEDV, left ventricular end-diastolic volume; LVEDV/BSA, left ventricular end-diastolic volume indexed for BSA; LVESV, left ventricular end-systolic volume; LVESV/BSA, left ventricular end-systolic volume indexed for BSA; LVEF, left ventricular ejection fraction; cm, centimeters; mm, millimeters; mmHg, millimeters of mercury; bpm, beats per minute; mL, milliliters; dL, deciliters; mg, milligrams; pg, picograms; ng, nanograms; min, minutes.

**Table 2:** Comparison of cardiometabolic parameters between groups, by TA.

	TA ≥ 70% (*n* = 26)				TA < 70% (*n* = 17)	
**(a) CV Parameters**	**Pre-Training T0**	**Post-Training T1**	***p*** -Value *	**Pre-Training T0**	**Post-Training T1**	***p*** -Value *
HR (bpm)	68.6 ± 8.3	62.6 ± 5.7	0.003	66.6 ± 6.8	62.3 ± 5.5	0.002
RWT	0.42 ± 0.06	0.38 ± 0.04	0.01	0.37 ± 0.05	0.38 ± 0.04	0.52
E/A ratio	0.92 ± 0.3	1.1 ± 0.24	0.01	0.93 ± 0.26	0.98 ± 0.28	0.53
E/e’	7.3 ± 1.8	6.1 ± 1.6	0.01	6.1 ± 1.6	6 ± 1.4	0.81
**(b) Metabolic Parameters**	**Pre-Training T0**	**Post-Training T1**	***p*** -Value *	**Pre-Training T0**	**Post-Training T1**	***p*** -Value *
HC (cm)	100.9 ± 7.3	98.8 ± 7.1	0.003	103.7 ± 9.4	101.6 ± 7.6	0.005
VO2 max (mL/kg/min)	26.7 ± 4.8	28.4 ± 4.8	0.007	23.7 ± 6	26 ± 6.6	0.21
Cortisol (pg/mL)	107.8 [93.3–159.2]	91.4 [65.5–116.5]	<0.001	98 [74.3–126.5]	94.6 [73–128.2]	0.25
Leptin (ng/mL)	45.7 [22.5–70.2]	37.6 [10.1–62]	<0.001	44.3 [28–66.8]	41 [23.3–47.1]	< 0.001
Cortisol/DHEA-S	148.5 [77.2–244.3]	130.5 [39.1–221.7]	0.016	137 [72–208.7]	124 [81.2–174.6]	0.20
Serum glucose (mg/dL)	79 [77–83]	75.5 [70–81]	0.02	81 [77–84]	78.5 [73–80]	0.66

Legend: Data are described as mean ± SD or median (Q1, Q3) for quantitative variables; *: Student’s T-Test or non-parametric T-test. Abbreviations: HR, heart rate; RWT, relative wall thickness; HC, hip circumference; VO2 max, maximal oxygen uptake; DHEA-S, dehydroepiandrosterone sulphate; bpm, beats per minute; cm, centimeters; mL, millimeters; kg, kilograms; min, minutes; pg, picograms; ng, nanograms; mg, milligrams; dL, deciliters.

**Table 3:** Analysis of cardiometabolic parameters, by TA.

**(a) Cardiovascular Parameters**	**TA < 70% (*n* = 17)**	**TA ≥ 70% (*n* = 26)**	***p*-Value**
RWT	0.06 ± 0.03	0.15 ± 0.02	0.038
E/e’	0.09 ± 0.09	(−)0.35 ± 0.06	0.039
**(b) Anthropometric and Laboratory Parameters**	**TA < 70% (*n* = 17)**	**TA ≥ 70% (*n* = 26)**	***p*-Value**
BMI	0.02 ± 0.00	(−)0.01 ± 0.001	0.046
Weight (kg)	0.02 ± 0.00	(−)0.01 ± 0.001	0.046
Cortisol (pg/mL)	0.46 ± 0.09	(−)0.21 ± 0.07	0.041

Abbreviations: TA, total adherence; RWT, relative wall thickness; BMI, body mass index; kg, kilograms; pg, picograms; mL, milliliters.

**Table 4:** Correlates of RWT and cortisol delta absolute change.

**DELTA RWT**	**Beta Coeff.**	**95% CI**	***p*-Value**
TA ≥ 70%	0.089	0.012, 0.16	0.023
BOUT10	(−)0.0004	(−)0.001, 0.0004	0.33
SedT	(−)0.006	(−)0.0003, 0.0001	0.59
**DELTA Cortisol**	**Beta Coeff.**	**95% CI**	***p*-Value**
TA ≥ 70%	(−)0.20	(−)0.43, 0.025	0.045
BOUT10	(−)0.001	(−)0.004, 0.0016	0.40
SedT	(−)0.0001	(−)0.0008, 0.0006	0.78

Abbreviations: RWT, relative wall thickness; TA, total adherence; BOUT10, 10-min bouts of spontaneous moderate-to-vigorous physical activity; SedT, sedentary time.

**Table 5:** Armband analysis of daily spontaneous PhA before (T0) and after training (T1).

	Pre-Training T0	Post-Training T1	*p*-Value
DEE avg (min/die)	2231.73 ± 297.8	2418	<0.001
MET avg	1.4 ± 0.26	1.47 ± 0.38	0.32
STEPS (avg, min)	11074 [9222, 13868]	12669 [8437.5, 14640.5]	0.20
LIPAT (1.5–3 Mets; avg, min)	325.5 [251, 439]	358 [254, 410]	0.50
MIPAT (3–6 Mets; avg, min)	5070 [3960, 8910]	4740 [3360, 9720]	0.80
SedT (min)	630 [535, 714]	591 [454, 669]	0.20

Abbreviations: DEE, average of daily energy expenditure; MET, metabolic equivalent; LIPAT, low-intensity physical activity; MIPAT, moderate-intensity physical activity; SedT, sedentary time; min, minutes; avg, average.

## Data Availability

The data presented in this study are available on request from the corresponding author.
